# Supplementary material for: Tsg101 chaperone function revealed by HIV-1 assembly inhibitors
Source: Nat Commun. 2017 Nov 9;8:1391. doi: 10.1038/s41467-017-01426-2 (PMC5680296; doi:10.1038/s41467-017-01426-2)
Supplement: Supplementary file 1 — Supplementary Information [file 41467_2017_1426_MOESM1_ESM.pdf]

Supplementary Information

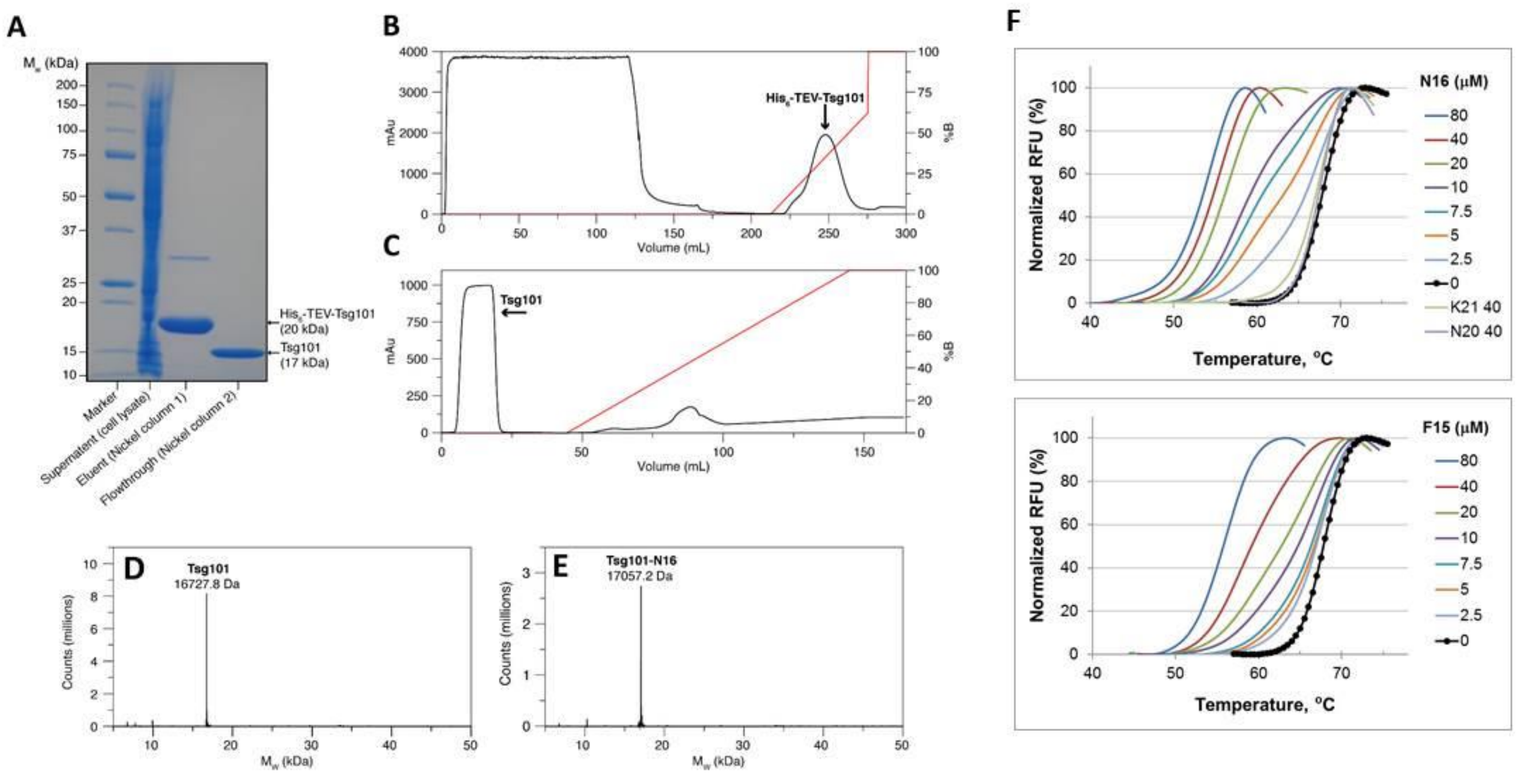

Supplementary Figure 1. The ubiquitin E2 variant (UEV) domain of Tsg101 was purified by attaching it to an N-terminal His_6 tag with a Tobacco Etch Virus nuclear-inclusion-a endopeptidase (TEV) cleavage site and isolating the tagged protein through two consecutive nickel columns. The first nickel column bound the Tsg101 with the His_6 tag. Following cleavage of the His_6 tag, Tsg101 flowed straight through the second column, removing any impurities from the cell lysate that also bound to the column in step 1, as well as the TEV protease enzyme. **a,** SDS-PAGE showing the cell lysate, the bound fractions from nickel column 1 (including His_6-TEV-Tsg101 and some impurities) and the flow-through from nickel column 2 (pure Tsg101). **b**, The FPLC trace for nickel column 1 (red line, imidazole gradient). **c**, The FPLC trace for nickel column 2 (red line, imidazole gradient). **d**, LC-MS for free Tsg101 (cleaved). **e**, LC-MS for Tsg101-N16 (increased mass indicates N16 binding). **f**, Dose-dependence of Tsg101 normalized melting curves for N16 (top) and F15 (bottom). At same concentration, N16 shifts the melting curve to a greater extent to the left than F15. Specifically, at 20 μM, N16 caused a 12˚C T_m shift while F15 gave rise a 5˚C T_m shift. This difference correlated with the potency observed in the cell-based assay. The plot for N16 contains two control compounds, K21 and N20, which have no effect at 40 μM on the Tsg101 melting curve.

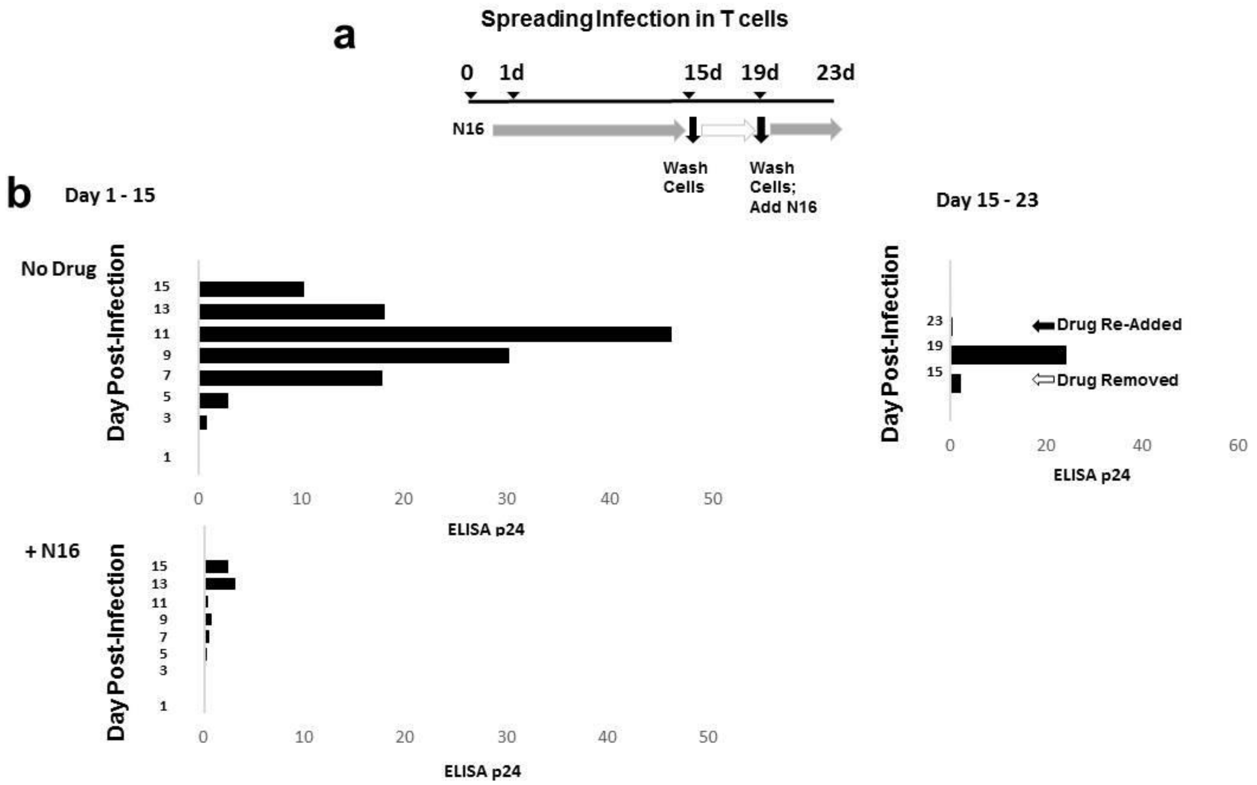

Supplementary Figure 2. N16 inhibition of HIV-1 transmission in a spreading infection in Jurkat cells. **a**, Schematic diagram summarizing experimental protocol. Jurkat cells (triplicate samples of 5 x 10^5/well) were incubated for 2 hr with HIV-1 NL4-3 in treatment media containing 50 µM N16 or control vehicle (DMSO). At the end of this period, unbound virus was removed by centrifugation and the cells washed once, resuspended in fresh control or treatment media and returned to the 37˚C incubator. For the next 15 days, tissue culture media was removed daily by centrifugation and saved for virus measurement by p24 capture ELISA and cells were replenished with fresh control or N16 treatment media. **b**, ELISA readings. In control media (containing DMSO), virus replication peaked at day 11; in the presence of N16, production peaked at 13 days. A comparison of peak values indicated that N16 reduced virus production 15-fold. Cells were monitored periodically for viability by Trypan Blue assay. To test their ability to produce virus after sustained drug exposure, at day 15 the N16-treated cells were washed, fresh media without inhibitor was added and the cells were incubated 4 days longer (to day 19). The supernatant was collected, the cells were washed again and then incubated for another 4 days in media with inhibitor, adding fresh inhibitor daily. The final supernatant was collected on day 23. The virus level surged by 10-fold when N16 was removed indicating that the observed inhibition was not attributed to irreversible cell toxicity. It then plummeted by 50-fold upon re-addition of N16, indicating that the virus was still susceptible. Thus, the cells maintained the ability to produce virus and the virus in the population at 15 days was essentially as susceptible to N16 as the initial virus population used to infect the Jurkat cells. Number of independent trials: $n = 2$.

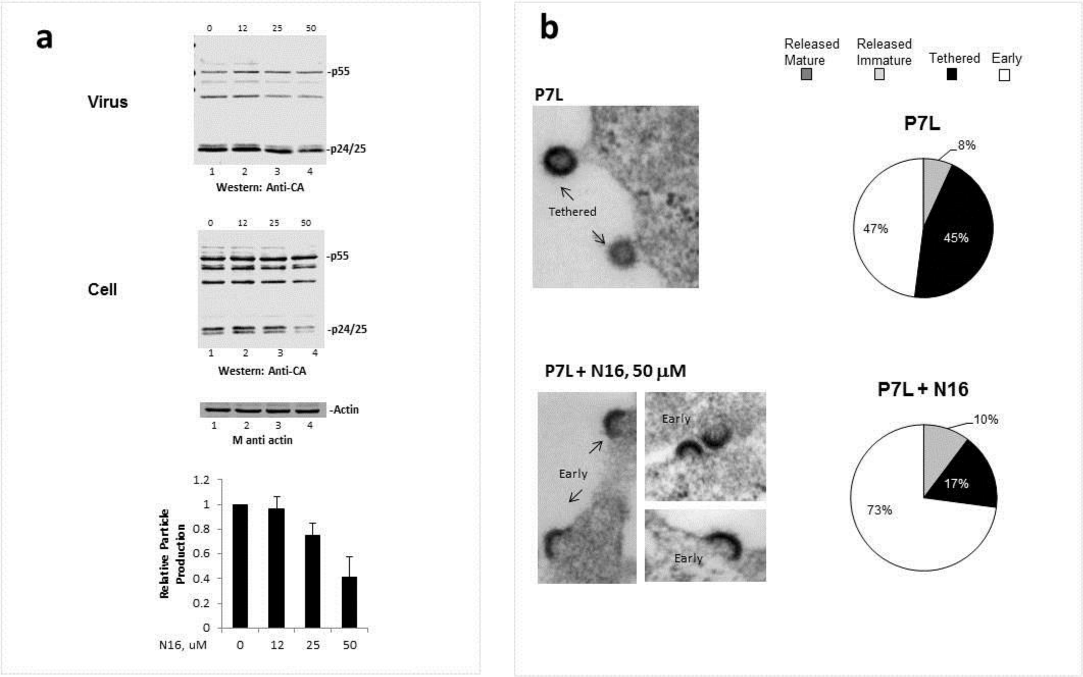

Supplementary Figure 3. N16 inhibits residual release of P7L mutant virus. 293T cells were transfected with DNA (P7L mutant pNL4-3 ΔEnv + HIV-1 IIIB-Env) at 6 hrs after N16 treatment. After 24 hr, tissue culture media was removed for VLP isolation and cell lysates were prepared. **a**, Western analysis of virus particles released in tissue culture media (top) and cell-associated Gag and actin (middle) and virus production normalized to the mock-treated control (bottom). Number of independent assays: $n = 3$. **b**, Quantitative analysis of budding morphologies in cells exposed to either DMSO carrier or 50 µM N16. Change in 'Early' bud detection: Chi-square p value <0.0001.

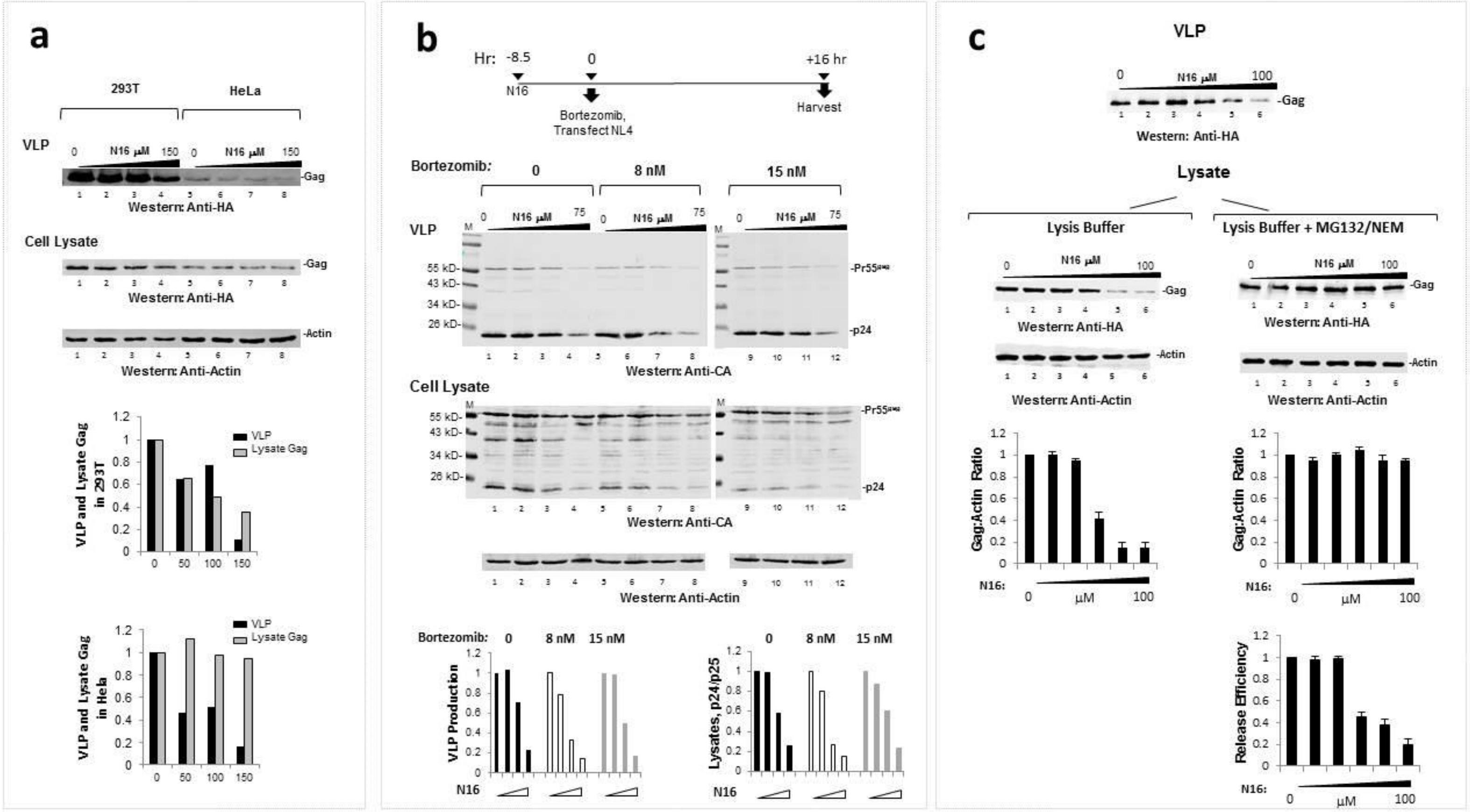

Supplementary Figure 4. **a**, Effect of N16 on Gag VLP production and Gag steady-state in 293T and HeLa cells. Top: Cells treated with 0, 50, 100, and 150 μM N16 were transfected with DNA encoding Gag-HA. At the end of the treatment period, tissue culture media was removed for VLP isolation. Cells were suspended in lysis buffer. Blots were probed for Gag-HA and actin. Bottom: Quantitative analysis. Relative effects of N16 on VLP and S1 Gag. **b**, Effect of *in-cellulo* bortezomib on N16-mediated interference with VLP production and Gag steady-state. Top: Schematic diagram summarizing experimental protocol**.** Middle: Western analysis of isolated virus and cell lysates. Cells were exposed to 0, 25, 50, and 75 μM N16 for 8 to 9 hr prior to transfection with DNA encoding pNL4-3ΔEnv and bortezomib at 0 (lanes 1-4), 8 (lanes 5-8) or 15 (lanes 9-12) nM. 16 hr later, the media was removed for VLP isolation and the cells were washed and lysates prepared. The wash and cell lysis buffers contained bortezomib at 10 nM as it is a reversible inhibitor. Bottom: Quantitative analysis. **c**, Effect of *ex-cellulo* treatment with proteasome inhibitor MG132 and NEM on N16-mediated interference with Gag steady-state. Top: Cells treated at the indicated concentration were transfected with DNA encoding Gag-HA. At the end of the treatment period, tissue culture media was removed for VLP isolation. Middle: Cells were either suspended in lysis buffer (left) or incubated 1 hr further in media containing 25 μM MG132 and 10 μM NEM and then suspended in lysis buffer containing 25 μM MG132 and 10 μM NEM. Blots were probed for Gag-HA and actin. Bottom: Quantitative analysis, Ratio of Gag to actin normalized to the mock-treated control (0 μM N16). VLP release efficiency was determined as the ratio of [Gag signal in VLP]/[Gag signal in VLP + Gag signal in cell lysate prepared with MG132/NEM]. Values were normalized to that of the mock-treated control. **a**, n = 2; **b**, n = 1; ***c***, n = 2; error bars represent 1 SD for the assay values (normalized to 0 uM N16).

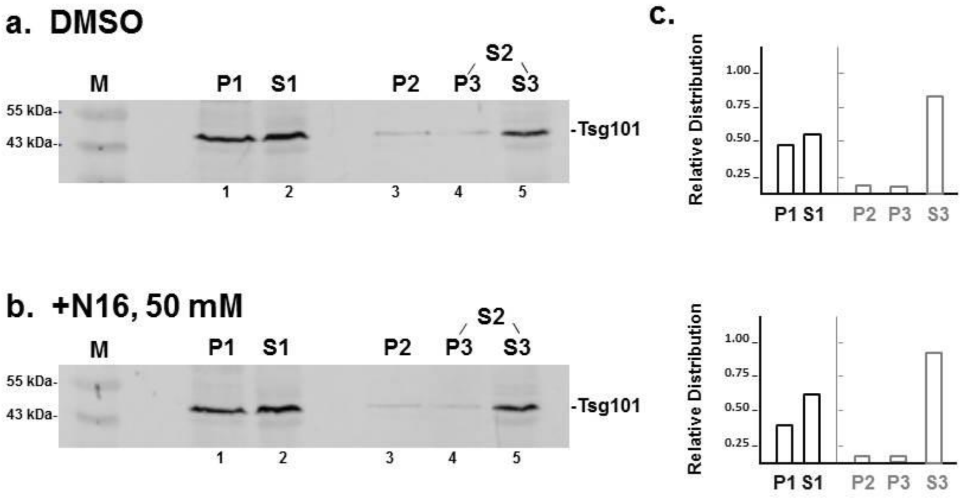

Supplementary Figure 5. Localization of endogenous Tsg101 +/- N16. Upon reaching the typical confluency used in experiments above, the tissue culture media was removed from 10 cm plates of 293T cells and replaced with media containing either 50 μM N16 or the DMSO vehicle. After a 24 hr treatment period, cells were harvested and suspended in hypotonic buffer, Dounce homogenized and the homogenate subjected to differential centrifugation to obtain subcellular fractions. Homogenates were centrifuged at 1,000xg to remove nuclei and cell debris (P1) from a post-nuclear supernate (S1) which was further centrifuged at 27,000xg to pellet endosomes and lysosomes (P2) yielding a supernate (S2) which was further centrifuged at 100,000xg to separate PM-derived microsomes and small vesicles (P3) from non-membrane-bound cytosolic proteins (S3). Pellet fractions were resuspended in RIPA to the original homogenate volume. Fractions obtained from DMSO- (**a**) and N16- (**b**) treated cells were analyzed by Western for endogenous Tsg101. Semi-quantitative analysis (**c**) showed an essentially identical localization for Tsg101 in control- and drug-treated cells. $n = 2$.

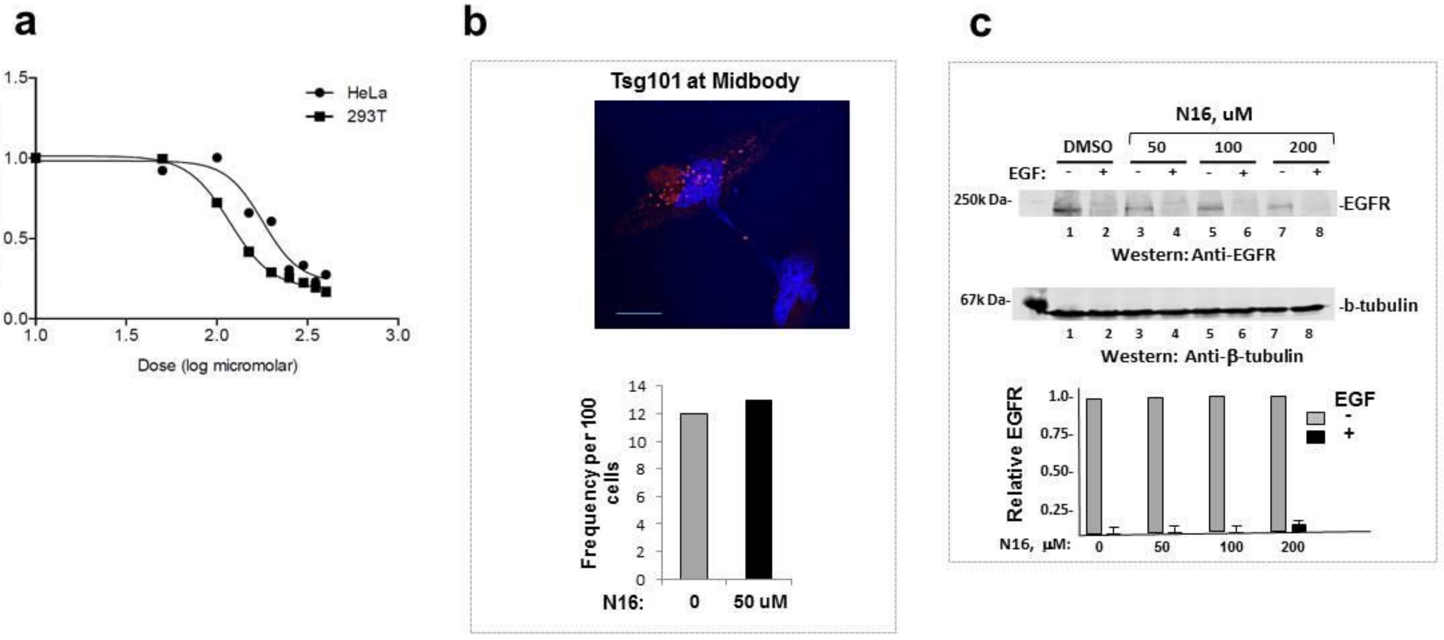

Supplementary Figure 6. N16 inhibition is virus-specific. **a**, N16 IC_{50} for HeLa and 293T cells. At 95% confidence levels, the IC_{50} values for HeLa and 293T were 156 – 205 μM and 99.8 – 139.4 μM, respectively (Prism 6, Graph Pad Software Inc.). Values were calculated by measuring metabolic activity (WST-1 Assay, Roche Applied Science) after cells were grown for 24 hr in N16. Data points represent the mean of 2 assays, 3 time points each for each cell line. **b**, The frequency of cells exhibiting Tsg101 midbody localization in 200 cells sampled was determined. Values in the absence or presence of N16 (50 μM) were 12% and 13%, respectively). Scale bar = 10 microns. **c**, N16 effect on EGF-stimulated EFGR degradation. Cells maintained for 24 hr in media containing DMSO alone or DMSO and N16 were stimulated with EGF ligand for a 90 min period and then assessed for EGFR steady-state level by Western analysis. EGFR levels for unstimulated and stimulated sample pairs are shown. **a**, n = 2; **b**, n = 2; **c**, n = 2; error bars represent 1 SD for the assay values (normalized to -EGF).

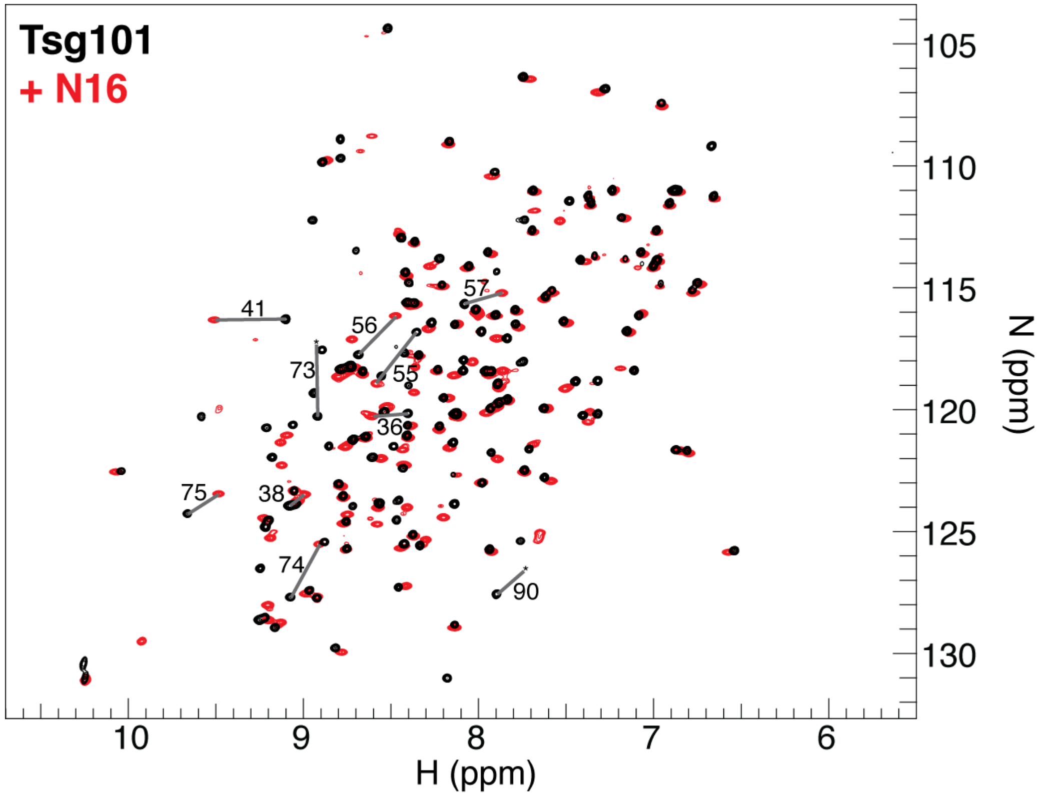

Supplementary Figure 7. HSQC spectra of the Tsg101 UEV domain in the presence and absence of N16. ^{15}N-Tsg101 UEV HSQC spectrum in the absence (black) and presence (red) of N16 (excess N16 and DMSO removed by ultrafiltration). The ten largest chemical shift perturbations are highlighted with residue numbers. Stars indicate the location of residues C73 and K90, which are broadened in the presence of N16.

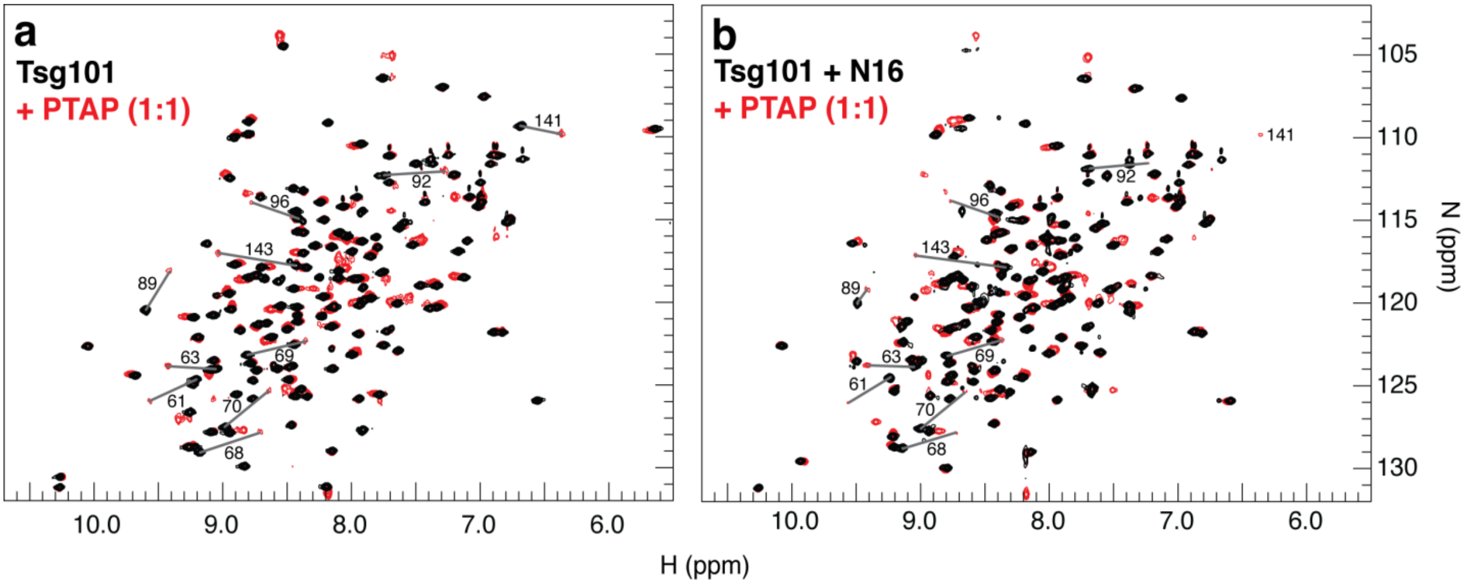

Supplementary Figure 8. HSQC spectra of the Tsg101 UEV domain in complex with the PTAP peptide, in the presence and absence of N16. **a**, ^{15}N-Tsg101 UEV HSQC spectrum in the absence (black) and presence (red) of PTAP at a 1:1 ratio. The ten largest chemical shift perturbations are highlighted with residue numbers. **b**, As in **a**, but with pre-incubation of N16.

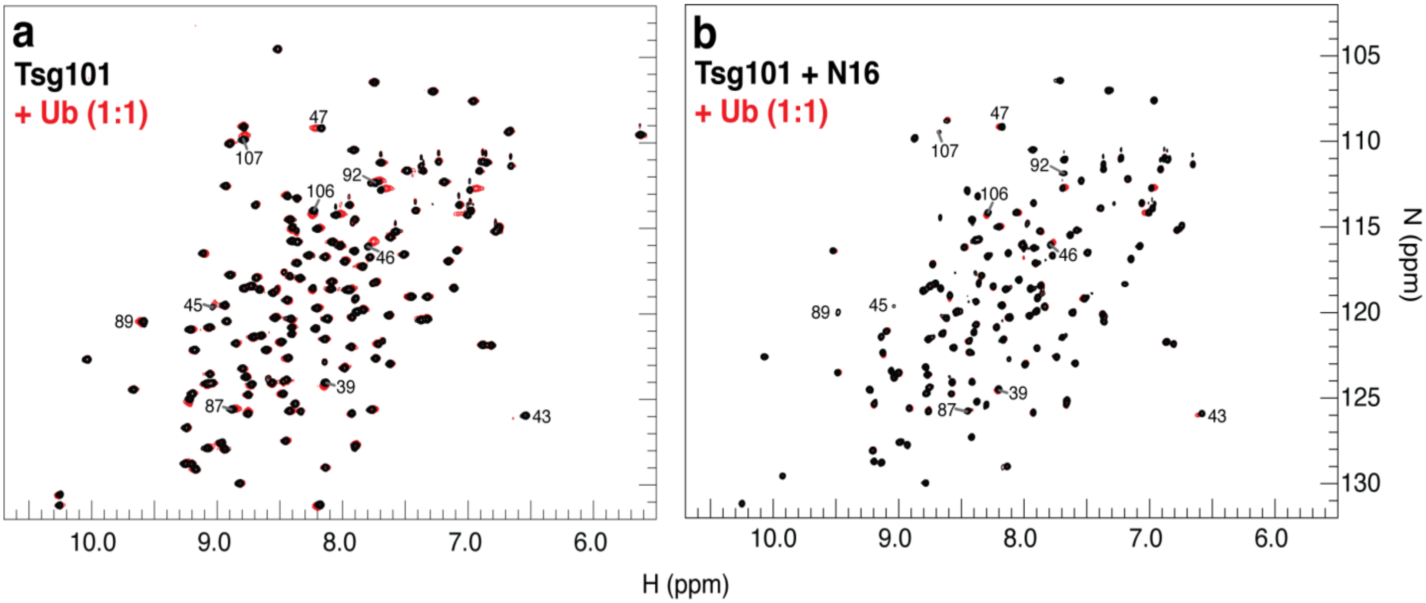

Supplementary Figure 9. HSQC spectra of the Tsg101 UEV domain in complex with ubiquitin, in the presence and absence of N16. **a**, ^{15}N-Tsg101 UEV HSQC spectrum in the absence (black) and presence (red) of Ub at a 1:1 ratio. The ten largest chemical shift perturbations are highlighted with residue numbers. **b,** As in **a**, but with pre-incubation of N16.

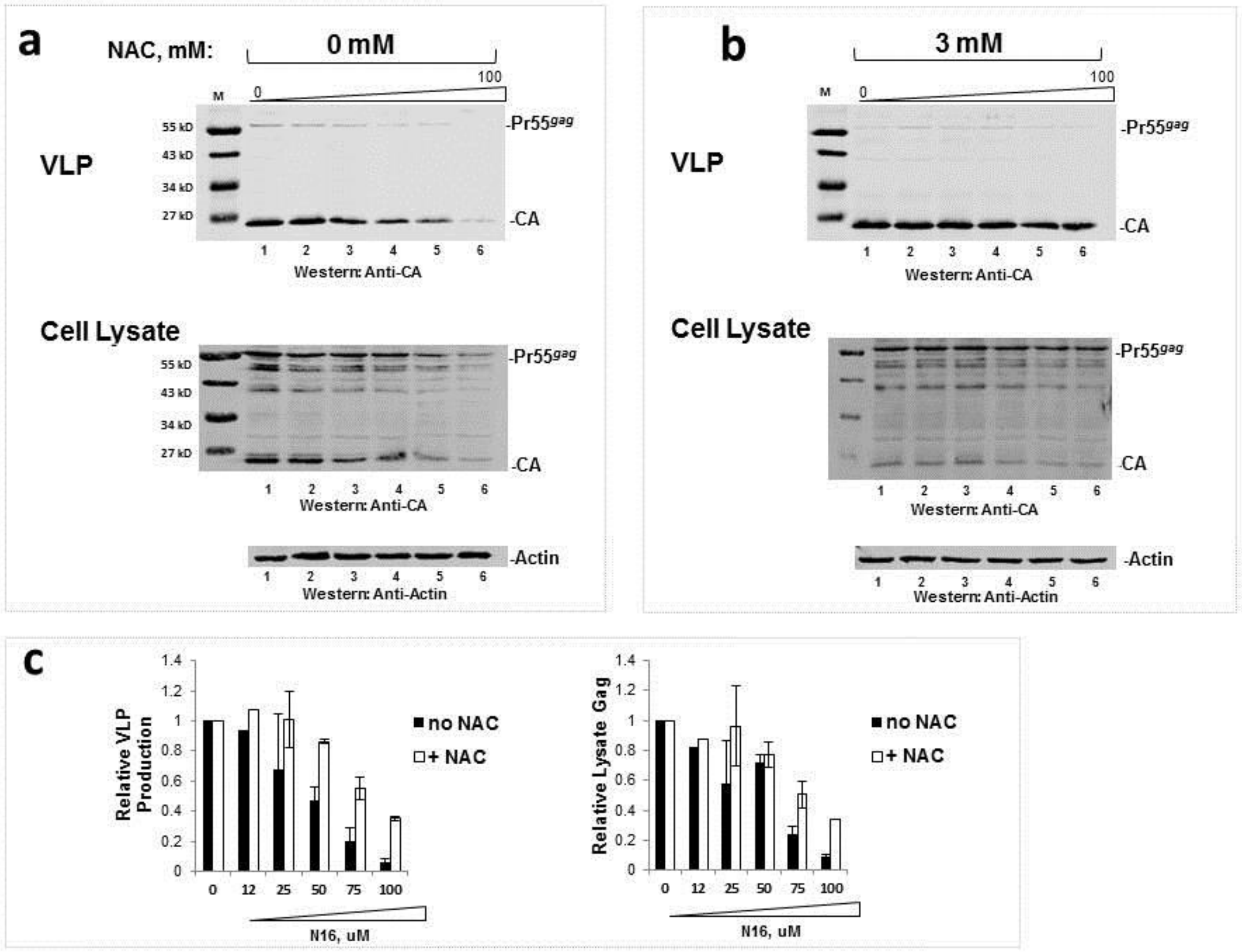

Supplementary Figure 10. Effect of N-acetyl cysteine (NAC) on N16-mediated inhibition of VLP production and intracellular Gag accumulation. Metabolic measurements (Roche WST-1 reagent) indicated that 293T cells were robust up to at least 4 mM NAC. A concentration of 3 mM NAC was therefore used to test the effect of the antioxidative reagent on the N16 inhibitory effect. N16 (0, 12, 25, 50, 75, and 100 μM) was added 8.5 hr prior to transfection with pNL4-3ΔEnv. Cells were harvested 16 hr later. **a,** Dose-dependent inhibition of VLP production (top) and intracellular Gag accumulation (Bottom) by N16. **b,** Suppression of the N16 inhibitory effect by 3 mM NAC. **c,** Quantitative analysis. *n* = 2; error bars represent 1 SD for the assay values (normalized to 0 uM N16).

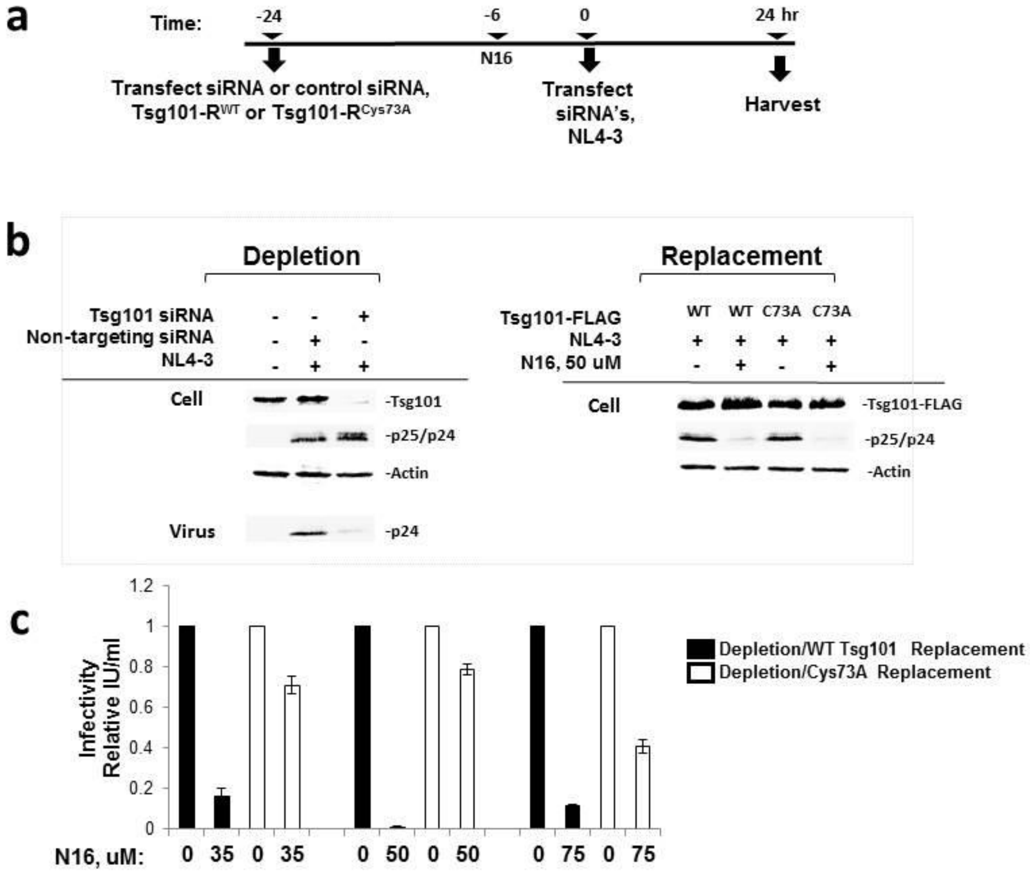

Supplementary Figure 11. N16 inhibition is suppressed by replacing endogenous Tsg101 with the Tsg101-C73A mutant. **a**, Schematic diagram summarizing experimental protocol. **b**, Western analysis showing Tsg101 steady-state level following depletion (left) or replacement (right). **c**, Quantification of released viral particles following replacement with WT (left) or C73A (right), replacement as measured by the MAGI assay. **a**, 293T cells were transfected with non-targeting siRNA or siRNA targeting Tsg101. Thirty minutes later, cells treated previously with the targeting siRNA were transfected with siRNA-resistant replacement Tsg101 constructs (R^{WT} or R^{C73A}). Eighteen hours later, all cells were treated with the DMSO control or with 50 μM N16. Six hours later, all cells were co-transfected with DNA (pNL4-3 ΔEnv + HIV-1 IIIB-Env) and additional siRNA. Tissue culture media was collected 24 hr post-DNA transfection, filtered and examined for virus production. **b**, The targeted siRNA reduced the steady-state level of Tsg101 in the cell lysate compared to the level in mock-treated cells or cells transfected with the non-targeting control siRNA. The specificity of the siRNAs was indicated by the finding that the actin level was not affected. **c**, In cells where siRNA-resistant WT Tsg101 was expressed following targeted siRNA depletion, N16 reduced the amount of infectious units in the MAGI assay. Less N16 inhibition was observed following replacement with the siRNA-resistant C73A Tsg101 variant. The results support the conclusion that C73 is a critical residue for the antiviral effect. (*n* = 2); error bars represent 1 SD for the assay values (normalized to 0 uM N16).

Figure 1

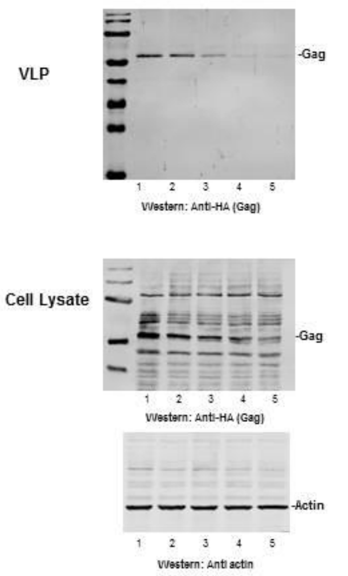

Figure 3a

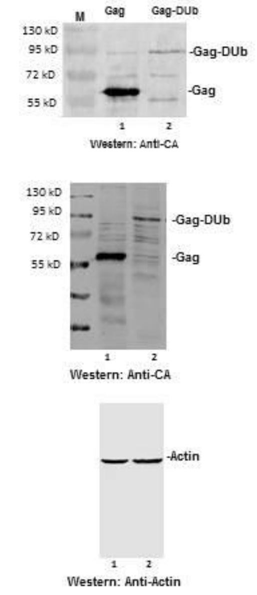

Figure 3b

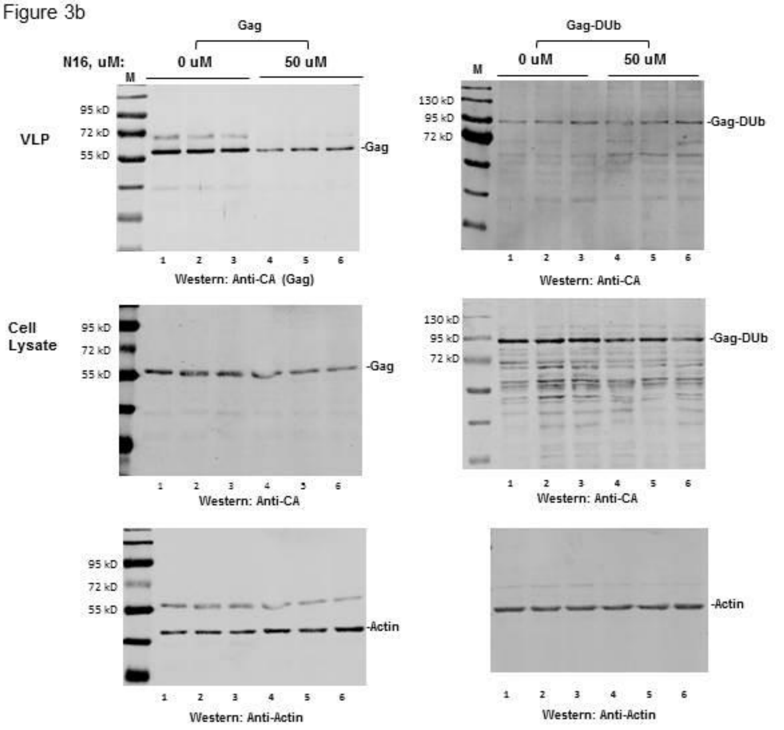

Supplementary Fig.3 Actin Gel

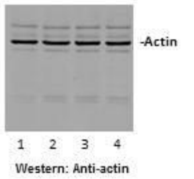

Supplementary Fig.4a

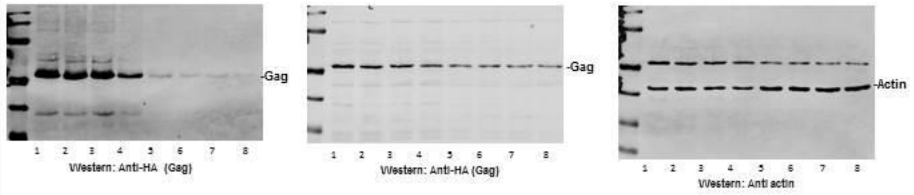

Supplementary Fig.4b

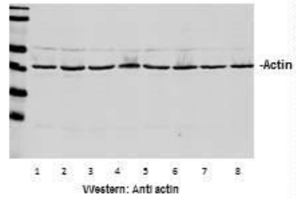

Supplementary Fig.4c

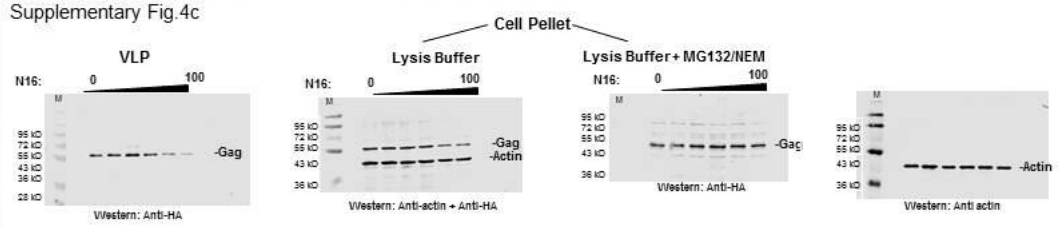

Supplementary Fig.5 a and b

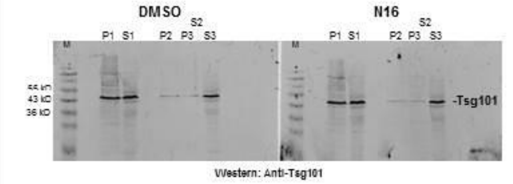

Supplementary Fig.6c

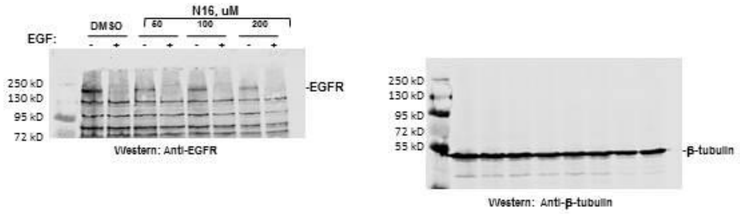

Supplementary Fig.10 a and b

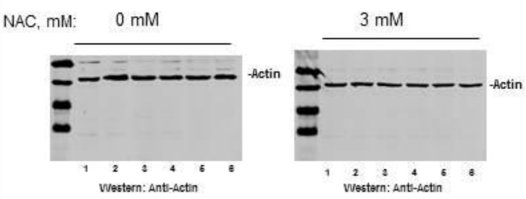

Supplementary Fig.11b

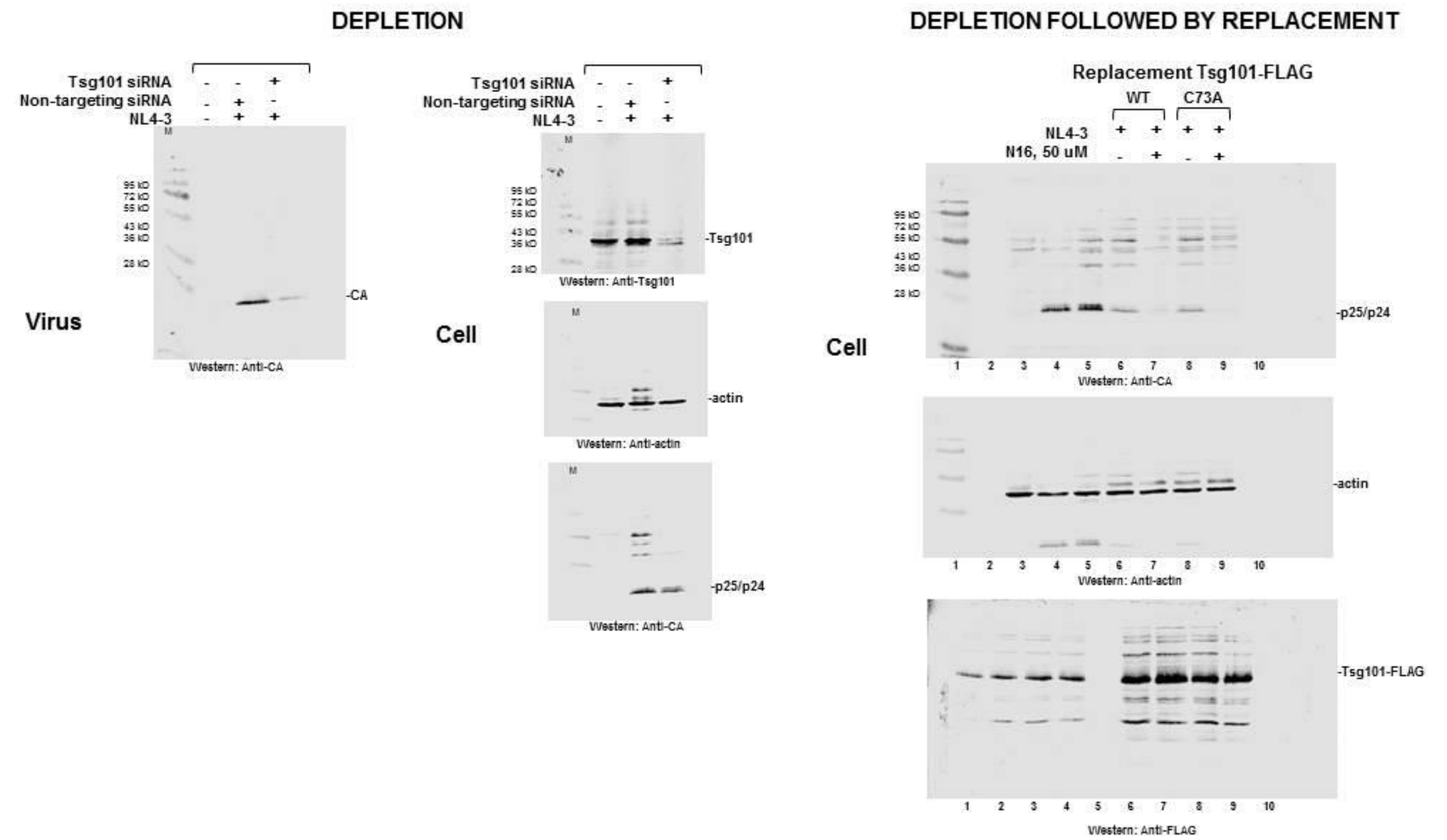

Supplementary Figure 12. Uncropped blot images used to compose Western result panels in indicated figures.
